# Molecular mechanisms and physiological functions of autophagy in kidney diseases

**DOI:** 10.3389/fphar.2022.974829

**Published:** 2022-08-11

**Authors:** Jingchao Yang, Longhui Yuan, Fei Liu, Lan Li, Jingping Liu, Younan Chen, Yanrong Lu, Yujia Yuan

**Affiliations:** Key Laboratory of Transplant Engineering and Immunology, NHC; Frontiers Science Center for Disease-Related Molecular Network, West China Hospital, Sichuan University, Chengdu, China

**Keywords:** autophagy, kidney aging, kidney fibrosis, acute kidney injury, chronic kidney disease

## Abstract

Autophagy is a highly conserved cellular progress for the degradation of cytoplasmic contents including micromolecules, misfolded proteins, and damaged organelles that has recently captured attention in kidney diseases. Basal autophagy plays a pivotal role in maintaining cell survival and kidney homeostasis. Accordingly, dysregulation of autophagy has implicated in the pathologies of kidney diseases. In this review, we summarize the multifaceted role of autophagy in kidney aging, maladaptive repair, tubulointerstitial fibrosis and discuss autophagy-related drugs in kidney diseases. However, uncertainty still remains as to the precise mechanisms of autophagy in kidney diseases. Further research is needed to clarify the accurate molecular mechanism of autophagy in kidney diseases, which will facilitate the discovery of a promising strategy for the prevention and treatment of kidney diseases.

## 1 Introduction

Autophagy, a term derived from Greek meaning “self-eating,” was first proposed by Christian de Duve in 1963, soon after discovered “dense bodies” in rat liver (Novikoff et al., 1956; Glick et al., 2010; Ueno and Komatsu, 2017). However, surprisingly little attention has been devoted to “autophagy” for nearly 30 years (Cao et al., 2021). In the early 1990s, comprehensive research on the autophagy spread after discovering the autophagic degradation of cytosolic components during the nutrient-deficient conditions in yeast (Takeshige et al., 1992). Next, genetic analysis in the field of autophagy bloomed, providing insights into the function and mechanism of autophagy (Lin et al., 2019). In 2016, the Nobel Prize in Physiology or Medicine was awarded to Yoshinori Ohsumi for his work in elucidating the basic mechanism and physiological relevance of autophagy in human diseases (Levine and Klionsky, 2017). Since then, an ever-expending list of studies have yield many important advances in the understanding the role of autophagy (Kaushal et al., 2020).

Although the classical model of autophagy generally occurs in a wide variety of cell stresses, such as starvation, inflammation, and other pathologic conditions, a basal level of autophagy has also emerged in physiological conditions to maintain homeostasis for intracellular recycling and metabolic regulation (Ravanan et al., 2017). With the development of research, autophagy is now widely implicated in numerous diseases progressions including renal injury, indicating that further investigation of their therapeutic potentials are warranted (Kaushal et al., 2020; Peters et al., 2020).

## 2 Overview of autophagy

### 2.1 Classification of autophagy

**FIGURE 1:**
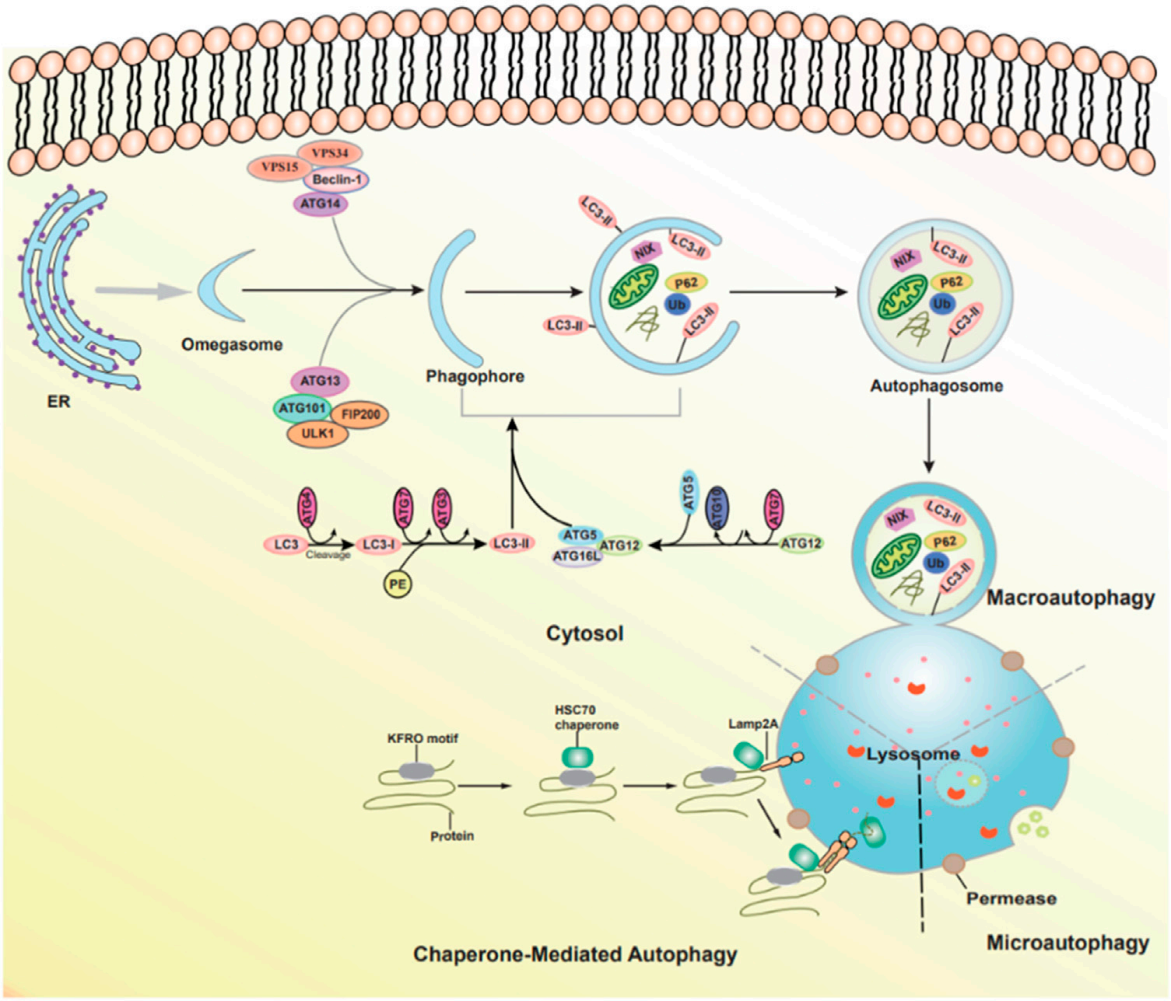
The types of autophagy and autophagic pathways. Three forms of autophagy can be identified: macroautophagy, microautophagy, and chaperone-mediated autophagy.

Autophagy is an essential cellular degradation process that delivers cytoplasmic components to lysosomes. Based on the type of cargo delivery to lysosomes, three forms of autophagy can be identified: macroautophagy, microautophagy, and chaperone-mediated autophagy. Macroautophagy (hereafter called autophagy) refers to the sequestration of cargo within an autophagosome, a double-membrane vesicular structure, which is formed after a phagophore (Feng et al., 2014; Li et al., 2020b). The autophagosome entraps target substrate to the lysosome and delivers its contents into lumen for degradation (Ravanan et al., 2017). Microautophagy involves the direct uptake of cytoplasmic contents by the invagination of lysosomes or endosomes, and lysosomal protrusion without the formation of autophagosome (Oku and Sakai, 2018). The third form of autophagy is chaperone-mediated autophagy (CMA), a set of process by which the proteins of KEFRQ-like motif are recognized by the cytosolic heat shock cognate 71 kDA protein and then translocated to lysosomes through interacting with lysosome-associated membrane glycoprotein 2A (LAMP2). Worthy of note, the progress is only for the proteins (Figure 1) (Kaushik and Cuervo, 2018; Al-Bari and Xu, 2020).

Additionally, autophagy is generally divided into two categories according to the different cargo selectivity: selective autophagy and nonselective autophagy (Nie et al., 2021). Selective autophagy refers to the degradation of a specific substrate with lysosome or vacuoles, depending on autophagy receptors, including mitophagy, lipophagy, pexophagy, etc. (Anding and Baehrecke, 2017; Li et al., 2021a; Nie et al., 2021). For mitophagy, the removal of organelles are accompanied by specific damaged mitochondria degradation. Similarly, for lipophagy and pexophagy, cytoplasmic lipid droplets and unwanted peroxisomes are removed from the cytoplasm by the autophagy pathway (Nie et al., 2021).Nonselective autophagy refers to the bulk transport of unspecific mixture of proteins and organelles (Knuppertz and Osiewacz, 2016).

### 2.2 Structures and functions of main molecules in autophagy

Autophagy is associated with a series of Autophagy-related genes (ATGs). The autophagic process can be divided into four steps. 1) In the initiation of autophagy: the multiprotein complex contains the serine/threonine protein kinase ULK1, FIP200, ATG13, and ATG101 (Galluzzi et al., 2017). 2) The formation of phagophore and autophagosome: the Class III phosphatidylinositol 3-kinase (PI3K) complex, including VPS15, VPS34, Beclin 1, and ATG14L; then the ATG2-WIPI complex and multiple ATG proteins are gathered to form isolation membrane. Two ubiquitin (Ub)-like conjugation systems ATG12-ATG5-ATG16L system and the microtubule-associated protein 1 light chain 3 (LC3) mediate the completion of the autophagosome (Li et al., 2020a; Tang et al., 2020). 3) Autophagosome-lysosome fusion: the movement of autophagosome to the lysosome is the successful prerequisite for fusions, including SNAREs, cytoskeleton components, and motor proteins (Lorincz and Juhasz, 2020). 4) Degradation and reformation of autolysosome: autolysosome is not permanent and disintegrates once autophagy is terminated, which is called autophagic lysosome reformation (ALR). During this process, lysosomal membrane proteins are recycled, and lysosomes regenerate through the reformation tubules and vesicles (Figure 1) (Yu et al., 2018).

## 3 Current perspectives of autophagy in kidney diseases

### 3.1 Mechanisms of autophagy regulation involved in kidney diseases

1) PI3K/AKT/mTOR; 2) AMPK/ULK1; 3) Sirt1/LC3; 4) PKCs; 5) ERK; 6) STING1.

#### 3.1.1 PI3K/AKT/mTOR

Phosphatidylinositol 3-kinases (PI3Ks) are a family of plasma membrane-associated lipid kinases. Based on their structural characteristics and specific substrates are normally divided into three classes: class I PI3Ks, class II PI3Ks, and class III PI3K (Yang et al., 2019; Xu et al., 2020b; Miricescu et al., 2021). Class II PI3Ks consist of a single catalytic Vps34 subunit. Early studies have shown that Vps34 induced autophagy *via* the mTOR pathway during nutrient deprivation (Backer, 2008). Moreover, mTOR is a negative modulator of autophagy. It is also known as the gated molecule of autophagy, which binds to the serine 757 of ULK1 and inhibits the AMPK-ULK1 interaction, leading to the inactivation of ULK1 and inhibiting autophagy (Miricescu et al., 2021).

PI3K is activated by a variety of extracellular stimuli, such as growth factors, hormones, and cytokines, which is a primary effector downstream of RTKs or GPCRs (Miricescu et al., 2021). Subsequently, these stimuli transduce into intracellular messages by phosphorylation of PtdIns (4,5) P2 (PIP2) to form PtdIns (3,4,5) P3 (PIP3) and recruit signaling proteins such as the serine/threonine kinase AKT. AKT is activated *via* two phosphorylation processes, phosphorylation of phosphoinositide-dependent-protein kinases 1 (PDK1) and phosphorylation of mTOR2 complex 2 (Miricescu et al., 2021). Collectively, the PI3K/AKT/mTOR pathway is engaged in cell survival and growth under a range of physiologic conditions.

Emerging data have reported that the PI3K/AKT/mTOR pathway is closely related to kidney diseases *via* regulating autophagy. Recently, Du et al. have revealed that the overexpression of protease activated receptors 2 (PAR2) in HK2 cells retards autophagy and leads to inflammation through activating PI3K/AKT/mTOR pathway. In mouse kidney, Nickle induced autophagy through activating AMPK and PI3K/AKT/mTOR pathways, which upregulated expression levels of p-AMPK, p-AKT and p-PI3K, to lead renal function injury (Yin et al., 2021). In summary, it is noteworthy that the PI3K/AKT/mTOR pathway has attracted much attention in regulating autophagy, suppressing the PI3K/AKT/mTOR signal pathway can enhance autophagy (Xu et al., 2020b).

#### 3.1.2 AMPK/ULK1

As a key energy sensor, AMP-activated protein kinase (AMPK) has an essential role in regulating cellular metabolism to maintain energy homeostasis (Hardie, 2007). ULK1 complex, a homologue of yeast ATG1, is pivotal for initiating autophagy. In particular, current researches declared that the regulation ATG1/ULK1 by mTOR and AMPK pathway were associated with kidney disease pathogenesis in diverse conditions, such as acute kidney disease (AKI) and diabetes mellitus. Under the nutrient-rich condition, activated mTOR phosphorylates ULK1 Serine 757 and inhibits the interaction between ULK1 and AMPK. Conversely, nutrient insufficiency, such as glucose deprivation, induces AMPK activation and directly activates ULK1 by phosphorylation of Ser 317 and Ser 777, promoting autophagy (Kim et al., 2011).

Currently, recent studies have yielded many important advances in the understanding of the connection between AMPK/ULK1 signaling pathway and kidney diseases. Shingo et al. confirmed that inhibition of autophagic activation in proximal tubules by impaired AMPK/ULK1 signaling and activated mTORC1 aggravated type 2 diabetes mellitus (T2DM)-induced renal injury (Muratsubaki et al., 2017). Upregulation of UCP1 could relieve lipid accumulation during cisplatin induced AKI mouse model and suppresses the disease progression by promoting the AMPK/ULK1/autophagy pathway (Xiong et al., 2021). Additionally, Liu et al. (2018) have estimated that inhibition of AMPK-ULK1-mediated autophagy mitigates renal aging by D-galactose. Similarly, Theodomir et al. used diabetic nephropathy (DN) mouse to identify that activated ULK1-mediated autophagy ameliorated fibrosis, inflammation, and oxidation, providing a potential therapy for DN (Dusabimana et al., 2021).

#### 3.1.3 SIRT1

Sirtuins comprise a conserved family of nicotinamide adenine dinucleotide (NAD+)-dependent histone deacetylases. SIRTI is the most extensively studied, which functions through deacetylating histones and non-histone proteins such as forkhead transcription factors (FOXOs) and p53 (Sosnowska et al., 2017). In addition, Lee et al. (2008) have demonstrated that SIRT1 is an essential regulator of autophagy, interacting with autophagy-related genes such as Atg5, Atg7, and Atg8. In addition, Huang et al. (2015) revealed that an SIRT1-mediated deacetylation of LC3, which interacted with other autophagy factors, playing a central role in autophagy. Similarly, Lee et al. (2008) demonstrated that SIRT1^−/−^ mice led to the accumulation of damaged organelles and disorder of energy metabolism, which were similar to Atg5 ^−/−^ mice. These studies indicated that the main function of SIRT1-mediated deacetylation could affect autophagic degradation.

Growing body of evidence indicates that SIRT1 links to kidney pathology. SIRT1 is highly expressed in medullary tubular cells and podocytes in the kidney (Morigi et al., 2018). In diabetic kidney disease, metformin relieved oxidative stress and enhanced autophagy *via* the AMPK/SIRT1 Foxo1 pathway (Ren et al., 2020). Also, Sun et al. (2021) showed that SIRT1 upregulation could ameliorate sepsis-induced acute kidney injury (SAKI) via deacetylating p53 to promote autophagy.

#### 3.1.4 PKCs

Protein kinase C modulates other proteins through phosphorylating serine and threonine amino acid residues (Wang et al., 2018), which contributes to maintaining cellular homeostasis through autophagy. However, uncertainty remains as to the association of PKC and autophagy (Wang et al., 2018). For example, Zhang et al. (2009) demonstrated that suppression of PKC significantly reduced oridonin-induced autophagy, concomitant with increased apoptosis, but these results were ameliorated by the PKC activator. Conversely, Jiang et al. (2010) showed that activation of PKC suppressed the autophagy progression of LC3I to LC3II during starvation or presence of rapamycin.

Concerning kidney diseases, a recent study has unveiled that PKC *δ* is a negative regulator of autophagy both *in vivo* and *vitro* cisplatin models, and PKC *δ* inhibitors protect kidneys during cisplatin treatment at least in part by facilitating autophagy (Zhang et al., 2017). Similarly, Xue et al. (2018) have confirmed that PKCα, as one of the significant sub pathways of mTORC2, mediates TGFβ1-inducing fibroblast activation and contributes to kidney fibrosis by upregulating autophagy flux.

#### 3.1.5 ERK

ERK, an extracellular signal-regulated protein kinase, is involved in many cell functions such as apoptosis, autophagy, and senescence (Cagnol and Chambard, 2010; Deng et al., 2021). However, the role of ERK pathway in kidney injury is still controversial (Cagnol and Chambard, 2010). On the one hand, Wu et al. (2017) reported that renalase could retard kidney fibrosis by inhibition of the ERK pathway. Similarly, Weng et al. (2020) demonstrated that interleukin (IL) -17A increased fibronectin production in human renal proximal tubular cells or renal fibroblasts and activated (ERK) 1/2 signaling pathway, whereas were ameliorated by ERK inhibitor U0126. On the other hand, recent work verified that geniposide (GEN) mitigated lipopolysaccharide (LPS)-induced apoptosis of podocytes by upregulating Ras/Raf/MEK/ERK-mediated autophagy (Li et al., 2019b).

#### 3.1.6 STING1

STING1, an evolutionarily conserved transmembrane protein, is located to the endoplasmic reticulum (ER) membrane in immune and non-immune cells. As an adapter protein, STING1 can produce type I interferons (IFNs) and proinflammatory cytokines in response to immune response and inflammation (Zhang et al., 2021). The mechanism of STING1-mediated autophagy is a noncanonical process that requires specific signals and regulators. STING1 can be directly activated by cGMAP, and leaves from ER to ERGIC, which acts as the source of membrane for autophagosome biogenesis. In addition, ATG5, ATG7, and WIPI2 are required for STING1-mediated autophagosome formation. RAB7 contributes to the transport of STING1 to lysosome from autophagosome and endosome. Under the condition of infection, STING1 not only as a regulator of autophagy, but also an autophagy substrate (Zhang et al., 2021).

**FIGURE 2:**
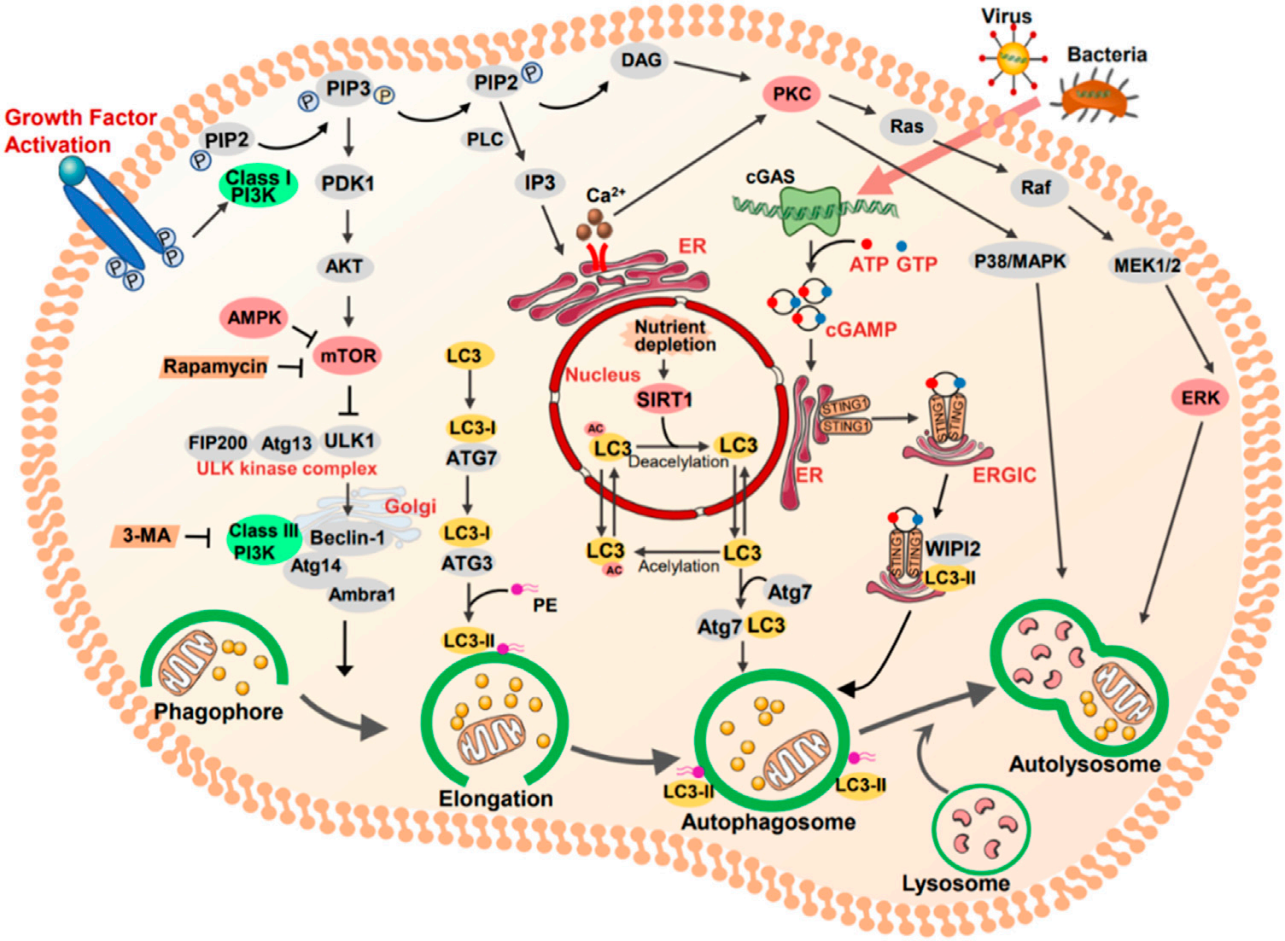
The key targets and signal pathways involved in autophagy. Class I PI3K (PI3K) is activated by growth factor, which as a primary effector downstream of RTKs or GPCRs. Subsequently, these stimuli transduce into intracellular messages by phosphorylation of PtdIns (4,5) P2 (PIP2) to form PtdIns (3,4,5) P3 (PIP3). In turn, the serine/threonine kinase AKT and other downstream are activated. Mammalian target of rapamycin (mTOR) can be activated by RACα serine/threonine protein kinase (AKT) and is a negative modulator of autophagy. AMP-activated protein kinase (AMPK) activated by several upstream kinases and inhibited mTORC1 to induce autophagy. Protein kinase C (PKC), as an autophagic regulator, modulating other proteins through phosphorylating serine and threonine amino acid residues. Ras/RAF/MER/ERK signaling functions downstream of PKC. Once MEK is activated, it phosphorylates ERK, and promotes autophagy. STING1 locates to the endoplasmic reticulum (ER) membrane in immune and non-immune cells, which activated by bacteria CDNS or CGAS-produced cGAMP. cGAMP binds to STING1, contributing to it translocated from ER to ERGLC, leading the formation of autophagosome.

Recent work confirmed that genetic deletion or pharmacological inhibitors of STING1 attenuated TFAM loss-mediated and FA-induced kidney fibrosis (Chung et al., 2019). In addition, Calio et al. (2021) demonstrated that STING1 is expressed in almost all renal perivascular epithelioid (PEC) lesions of kidney, providing the possible role of autophagy in PEC lesions of kidney (Figure 2).

### 3.2 The role of autophagy in kidney diseases

The kidneys are vital for blood filtration and osmotic balance. Dysregulation or failure of kidneys result in various renal pathologies, including AKI, chronic kidney disease (CKD), and renal fibrosis. Substantial evidence supports the vital role of autophagy in normal kidney functions (Hartleben et al., 2010). A thorough understanding of the regulatory mechanisms of autophagy in kidney diseases, may provide strategies and targets for therapeutic approaches.

#### 3.2.1 Autophagy in AKI

AKI is characterized by a rapid decline of kidney functions, coexisting with injury and death of the tubular epithelial cells, which may be contributed to CKD (Choi, 2020). Emerging evidence implicated that autophagy was upregulated in the kidney in the AKI induced by various insults such as renal ischemia-reperfusion (IR), sepsis, or nephrotoxis, playing a renoprotective role in kidney injury (Choi, 2020; Kaushal et al., 2020). Many studies revealed the role of autophagy during IR-induced AKI by utilizing conditional autophagy-deficient mice. Kimura et al. (2011) used a conditional Atg5 gene deletion model to verify the role of autophagy in AKI, and they confirmed that apoptosis of proximal tubular cells in autophagy-deficient mice were increased during I/R injury, concomitant with accumulation of p62 and ubiquitin-positive inclusions, compared with wild mice. These results suggested that autophagy maintained proximal tubular cell homeostasis and protected against IR. In addition, a study confirmed that autophagy was activated in the cisplatin-induced AKI model, whereas pharmacological inhibition of autophagy by 3-MA or shRNA knockdown contributed to tubular cell apoptosis (Periyasamy-Thandavan et al., 2008). Similarly, recent studies illustrated the role of autophagy in sepsis and AKI. Howell et al. (2013), utilizing lipopolysaccharide (LPS) - induced AKI, suggested that loss of autophagy in the kidney plagued recovery from septic AKI in aged mice; conversely, it can be rescued by restoring autophagic activity (Table 1).

**TABLE 1:** Effects of autophagy on AKI.

Animal model	Autophagy activity	Effect on AKI	References
Proximal tubules ablation of Atg5^flox/flox^	↓Autophagy	↑In AKI	Kimura et al. (2011)
Cisplatin-induced AKI	↑Autophagy	↓In AKI	Periyasamy-Thandavan et al. (2008)
LPS-induced AKI	↑Autophagy	↓In AKI	Howell et al. (2013)
Proximal tubules ablation of ATG7 ^flox/flox^	↓Autophagy	↑In AKI	Mei et al. (2016a)
Proximal tubules ablation of Atg5^flox/flox^	↓Autophagy	↑In AKI	Liu et al. (2012)
S3 segment of proximal tubules ablation of Atg5^flox/flox^	↓Autophagy	↓Tubular atrophy less interstitial fibrosis	Baisantry et al. (2016)
		↓inflammation at day 30 after I/R	

#### 3.2.2 Autophagy in CKD

##### 3.2.2.1 Autophagy in kidney aging

Renal aging is associated with characteristic structural and functional changes, including the susceptibility to acute kidney injury, the progression of CKD, and interstitial fibrosis (O’Sullivan et al., 2017). Glomerular podocytes and renal tubular cells are frequently implicated in renal senescence. On the one hand, podocytes are the structural constituent of the glomerular filtration barrier, playing a critical role in aged-related glomerular changes (Floege et al., 1997; Fang et al., 2020). In glomeruli, in the view that podocytes are terminally differentiated postmitotic cells, their capacities for regeneration are limited. Consequently, they require considerably efficient cellular mechanisms to maintain homeostasis. Recent theoretical development has revealed that autophagy was pivotal for podocytes, especially in maintaining homeostasis during kidney aging (Lenoir et al., 2016). Under basal conditions, podocytes have a high level of autophagy (Hartleben et al., 2010). In 20-to 24-month-old mice, the podocyte-specific ATG5 knockout mice displayed typical characteristics of aging cells, including mitochondrial damage, the accumulation of lipofuscin, and oxidized proteins (Hartleben et al., 2010). Concomitant with such results, podocyte-specific conditional knockout of Vps34 leads to early proteinuria and glomerular scarring, though defective autophagy was not primarily responsible for the severe phenotype caused by Vps34-deficient podocytes (Bechtel et al., 2013).

On the other hand, one study reported that renal tubulars cover over 90% of renal mass, interplaying between renal aging and fibrosis (Jin et al., 2019; Fang et al., 2020). The role of renal tubular is to reabsorb filtered solutes; the cells are inclined to consume more energy and accumulate oxidative damage during aging. In physiological conditions, the renal tubules show a low baseline turnover. However, a proliferative burst can be induced after damage (Schmitt and Melk, 2017). Earlier studies reported that p16^INK4a^ was found in almost cell types in kidney, but remarkably in tubular cells (Melk et al., 2004). Yamamoto et al. (2016) have uncovered what really matters in counteracting kidney aging is age-dependent high basal autophagy via mitochondrial quality control, as well as the relevance of a reduced level of upregulation of autophagic flux in response to metabolic stress in age-related kidney diseases. Also, one study has manifested that Brahma-related gene 1 (BRG1) potentiates tubular senescence and fibrotic responses *via* inhibition of autophagy through the Wnt/β-catenin pathway (Gong et al., 2021).

##### 3.2.2.2 Autophagy in kidney fibrosis

Kidney fibrosis, characterized by the accumulation of fibrous tissue, is a histological hallmark of CKD. Myofibroblasts are terminally differentiated cells, found in various pathologies that are considered to be the dominant collagen-producing cells at sites of fibrosis (Meran and Steadman, 2011). The origin of myofibroblasts in kidney fibrosis is heterogeneous, including endothelial cells, tubular epithelial cells, macrophages, pericytes, and BM-derived fibrocytes (Liu, 2011; El Agha et al., 2017). More recently, the potential role of tubular epithelial-mesenchymal transition (EMT) has been widely recognized in the development of fibrosis in chronic renal failure (Kriz et al., 2011). Generally speaking, the pathological processes of EMT in the renal tubulars have been described as the dedifferentiation of renal tubulars with loss of epithelial phenotype and acquisition of mesenchymal characteristics (Burns et al., 2007). During EMT in the renal tubulars, several steps appear necessary to complete this transformation including loss of cell contact and apical-basal polarity, disruption of the basement membrane, and the formation of enlarged spindle-shaped myofibroblast (Bedi et al., 2008; Burns and Thomas, 2010). So far, it remains controversial on the role of autophagy in kidney fibrosis was performed in models of fibrosis such as unilateral ureteric obstruction (UUO) or treatment with transforming growth factor (β1) TGF-β1 (Zhao et al., 2019).

##### 3.2.2.3 Pro-fibrosis effects of autophagy in the kidney

In mice subjected to UUO, Livingston et al. (2016) first reported that persistent autophagy in kidney proximal tubules. Of note, pharmacological inhibitors of autophagy or selective deletion of ATG7 in proximal tubules, reduced UUO-associated fibrosis, along with the attenuation of tubular atrophy, apoptosis, nephron loss, and interstitial macrophage infiltration (Livingston et al., 2016). The overexpression of WISP-1 increased the expression of LC3-II and Beclin-1 and exacerbated renal fibrosis in UUO models and TGF-β-treated tubular epithelial cells, which was abolished by anti-WISP-1 antibody and small interfering RNA (Yang et al., 2020). Furthermore, a recent study has demonstrated that C/BEP homologous protein (CHOP) plays a significant role in the progression of renal fibrosis, likely through autophagy and apoptosis, as evidenced by UUO-induced kidney fibrosis alleviated in the Chop^−/−^ than Chop^+/+^ mice (Noh et al., 2018). Similarly, Protein kinase Cα (PKCα), one of the major sub-pathways of mTORC2, plays a critical role in the relation between fibroblast activation and autophagy. A recent study further identified that enhancement of PKCα signaling promoted TGF-β1-stimulated fibroblast activation, which was reversed by PKCα inhibitor Go6979 and PKCα siRNA (Xue et al., 2018).

Notably, researchers suggested a connection between sustained activation of autophagy and lipid accumulation in tubular epithelial cells in the progression of kidney fibrosis. UUO-induced lipid accumulation in tubular cells was markedly reduced by pharmacological inhibition of autophagy 3-MA or CQ both *in vivo* and *in vitro* (Yan et al., 2018). In addition, under stress conditions, such as ischemia/reperfusion injury, autophagy is also implicated in kidney fibrosis. Compared with wild-type mice, conditional deletion of Atg5 in proximal tubular S3 segments presented with less tubular senescence, and interstitial fibrosis (Baisantry et al., 2016; Li et al., 2020b).

##### 3.2.2.4 Anti-fibrosis effects of autophagy in the kidney

Contrary to the above description, several studies have also demonstrated that autophagy has an antifibrotic role in kidney fibrosis. In a rat model of UUO, autophagy was induced in the obstructed kidney early after UUO, but inhibition of autophagy by 3-MA enhanced tubular cell apoptosis and tubulointerstitial fibrosis, indicating that autophagy might provide a protective role by suppressing tubular apoptosis (Kim et al., 2012). Similarly, Ding et al. (2014) constructed deletion of LC3B (LC3^−/−^) mice and Beclin 1 heterozygous (Beclin^+/−^) mice resulted in collagen deposition in the UUO model via further increased TGF-β expression. Additionally, one study further illuminated that autophagy in distal tubular epithelial cells played an antifibrotic role in renal tubulointerstitial by suppressing TGF-β and IL-1β pathways in UUO model (Nam et al., 2019). Furthermore, one study identified a relationship among autophagy, the cell cycle, and kidney fibrosis. In the UUO-induced kidney, genetic ablation of autophagy by proximal tubular epithelial cell-specific deletion of Atg5 observed severe interstitial fibrosis accompanied by markedly cell cycle arrest at the G2/M phase and robust COLI deposition. These results suggest that the regulation of cell cycle G2/M arrest by autophagy might be pivotal for the fibrogenic response (Li et al., 2016). Of note, another study showed that microtubule-associated protein 1S (MAP1S), as an autophagy activator, interacted with LC3 and involved in renal fibrosis. MAP1S deficiency in mice contributed to the accumulation of fibronectin and further aggravated the progression of renal fibrosis in aged mice. *In vitro*, MAP1S depletion in renal cells impaired the autophagy clearance of fibronectin and activated pyroptosis (Xu et al., 2016).

##### 3.2.2.5 Autophagy in kidney inflammation

**FIGURE 3:**
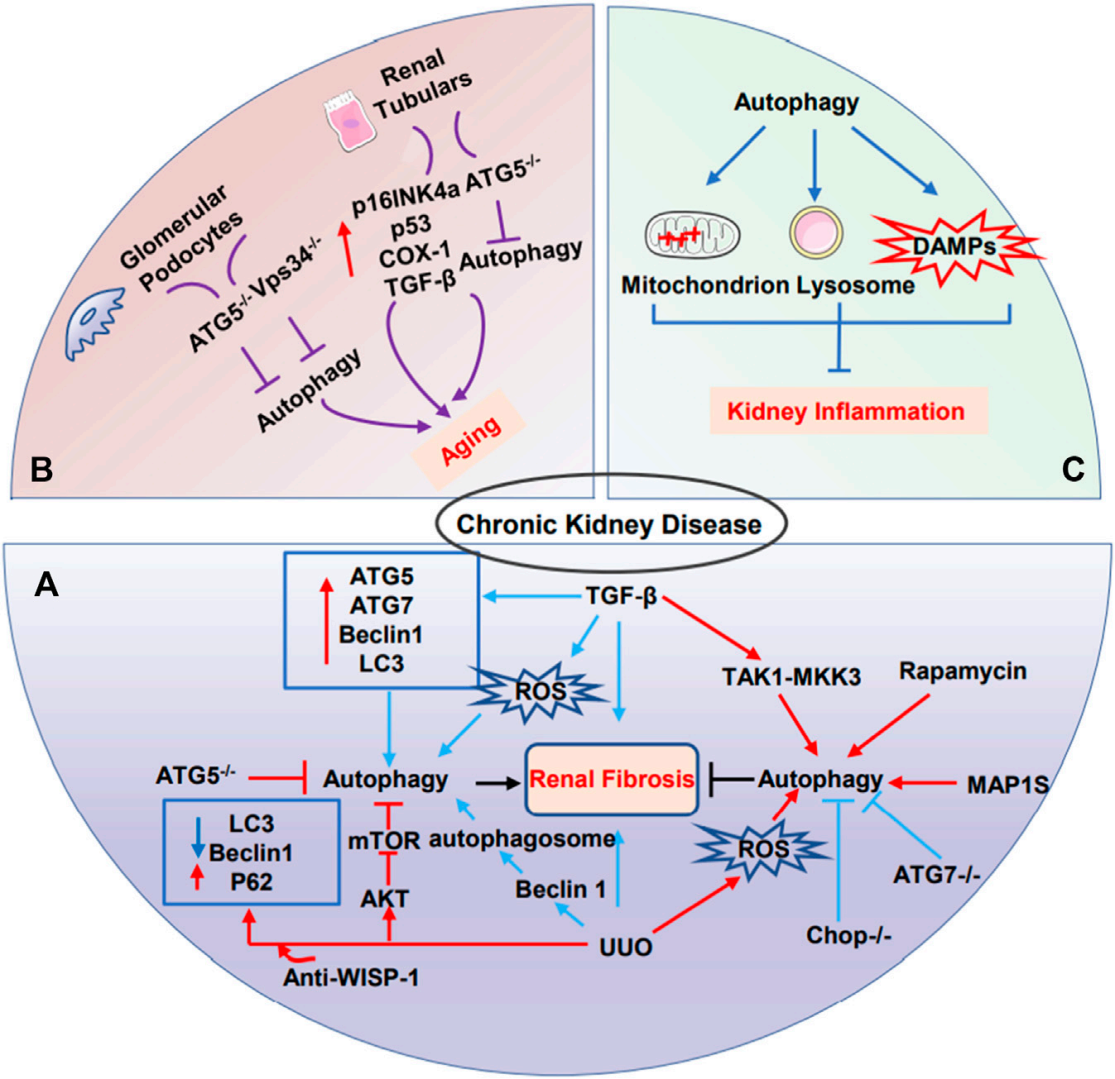
The role of autophagy in CKD. **(A)** The dual role of autophagy in renal fibrosis. **(B)** Glomerular podocytes and renal tubular cells are frequently implicated in renal senescence. Podocyte-specific ATG5 knockout mice or Vps34 knockout mice may lead to kidney aging. Renal tubulars-specific ATG5 knockout mice led to kidney aging. **(C)** Autophagy suppresses excessive inflammation through the clearance of damaged-mitochondrion, damaged-lysosome, and damaged-associated-molecular patterns to protect kidney.

Inflammation is the fundamental basis of most kidney disorders, including AKI, CKD, and aging (Kimura et al., 2017). A growing number of studies demonstrated the relevance of autophagy and kidney inflammation. Autophagy suppresses inflammation response *via* plaguing inflammasome production and interferon responses, accompanied by a reduction in damaged mitochondria, lysosomes, and damaged associated molecular patterns (DAMPs), which protects the kidney from injury (Kimura et al., 2017). Chronic inflammation may elicit renal aging, characterized by the accumulation of macrophages and lymphocytes in the kidney. It is noteworthy that chronic inflammation and kidney aging lead to a vicious cycle; on the one hand, chronic inflammation promotes renal aging by releasing pro-inflammatory factors, such as IL-1, IL-6, and TGF-β. On the other hand, these senescence cell results in secreting more inflammatory factors, which further aggravates fibrosis and CKD (Figure 3) (Bolignano et al., 2014).

#### 3.2.3 Autophagy for transition from acute kidney disease to chronic kidney disease

AKI is one of the global public health concerns associated with high morbidity, mortality, and medical costs both in the short- and long-term (Zuk and Bonventre, 2016; Cui et al., 2020). AKI may heighten the incidence of CKD and end-stage renal disease (ESRD). Notably, Tubular epithelial cell plays a central role both in AKI and CKD post-AKI (Basile et al., 2012; Humphreys, 2018; Li et al., 2019a). Nephrotoxicity, ischemia, sepsis, and hypoxia are the leading cause of AKI. Among these, ischemia injury and nephrotoxins are the two major causes of tubular damage (Basile et al., 2012). After damaged the kidney, tubular epithelial involves repair and regeneration. The process of repair may consist of complete repair and maladaptive repair. The repair can lead to complete renal recovery if the damage is mild. While severe injuries can result in fibrosis, facilitating progression to CKD. Therefore, maladaptive repair may link between AKI and CKD.

Recent theoretical developments have revealed tubular epithelial senescence and G2/M cell arrest are critical contributors to fibrosis progression (Ferenbach and Bonventre, 2015; Jiang et al., 2020). Senescence cells resist to apoptosis and secrete cytokines and chemokines, promoting the maintenance of a persistent inflammatory state.

In addition, senescence cells connect with increased expression of p16^INK4a^ and p21^WAF1^, facilitating growth arrest (Kuilman et al., 2010; Yang and Fogo, 2010). One study showed that G2/M-arrested proximal tubular cells triggered c-jun NH_2_-terminal kinase signaling (JNK), which contributed to upregulating profibrotic cytokine production (Yang et al., 2010).

Previously, researchers have identified the dynamic regulation of autophagy in postischemic kidneys and subsequent recovery. They utilized a new strain of autophagy reporter mice that expressed the differential pH sensitivities of red fluorescent protein (RFP) and enhanced green fluorescent protein (EGFP), contributing to advancing our understanding of autophagy. *In vivo* study confirmed that ischemia-reperfusion injury (IRI) induced autophagy in the proximal tubules at day 1 and autophagosome clearance at day 3 during renal recovery. Interestingly, inhibition of mTOR complex 1 led to autophagy persistent and decreased tubular proliferation, suggesting the role of mTOR in autophagy resolution during the renal repair (Li et al., 2014).

Moreover, as a cytoprotection protein, αKlotho is vital in tissue protection and regeneration (Hu et al., 2013; Shi et al., 2016). Researchers used two models: bilateral ischemia-reperfusion injury and unilateral nephrectomy plus contralateral ischemia-reperfusion injury and they found that αKlotho promoted kidney recovery, and ameliorated renal fibrosis by upregulating autophagy, but inhibition of αKlotho further potentiated collagen accumulation (Shi et al., 2016). Similarly, recent studies have elucidated that epithelial cell arrested at the G2/M phase was associated with synthesis and secretion of profibrotic cytokines by forming target of rapamycin-autophagy spatial coupling compartments (TASCCs). To investigate TASCC formation during the progression of CKD after AKI, they observed that TASCC was mainly expressed in PTCs and increased markedly from day 7 to day 21 after injury and maintained a high level on day 42 in fibrosis models, concomitant with up to 80% G2/M arrested cells. In addition, cyclin G1 played a key role during G2/M arrest and upregulated TASCC formation. Of interest, inhibition of TASCC formation mitigated fibrosis progression. Therefore, these results confirmed that G2/M-arrested proximal tubular cells might participate in forming TASCC *via* autophagy, promoting fibrosis during the maladaptive repair (Canaud et al., 2019; Tang et al., 2020).

## 4 Therapeutic application of autophagy enhancer in kidney diseases

Much of the research has examined that autophagy implicated in kidney diseases progressions and aging, as illustrated by AKI, CKD, etc. While autophagy is regulated by the mTOR-dependent and mTOR-independent pathways (Sarkar, 2013). Recent studies verified that autophagy activators mTOR inhibitors like sirolimus (rapamycin), and everolimus were used in clinical settings (Rini, 2008; Kajiwara and Masuda, 2016; Waldner et al., 2016). Additionally, luteolin, triptolide, dapagliflozin, cyclocarya paliurus triterpenic acids, rhein were demonstrated in experimental kidney diseases models (Li et al., 2017; Tu et al., 2017; Zhang et al., 2019; Jaikumkao et al., 2021; Xu et al., 2021). Moreover, mTOR-independent autophagy-inducing pharmaceutical agents such as trehalose, geniposide, sarsasapogenin, sulforaphane, salvianolic acid B, hyperoside, metformin, which were also verified in some kidney diseases models (Liu et al., 2018; Li et al., 2019b; Xu et al., 2020a; Fan et al., 2020; He et al., 2020; Lu et al., 2020; Zhu et al., 2020; Li et al., 2021b).

In an attempt to the analysis of autophagy *in vivo*, most studies used two types of mouse models, including “autophagy-deficient mice” and “autophagy-monitoring mice” (Kuma et al., 2017). Deletion of ATGs, such as ATG5, ATG7, and Beclin1, which facilitate to understanding of the physiological role of autophagy *in vivo* (Kuma et al., 2017). Similarly, autophagy-monitoring mice were used to monitor autophagic progress. Of note, transgenic mice systemically expressing EGFP-LC3 (GFP-LC3) have been widely used (Mizushima et al., 2004). However, clinal translation of autophagy regents remain arduous. On the one hand, ATGs-related genes mice by genetic techniques show various phenotypes, which are challenging to investigate the underlying mechanisms. On the other hand, the clinal regrets of autophagy might regulate autophagy progress and involve in other physiological progress. In the meantime, some drugs coexist with perilous side effects. Therefore, we indeed combine more precise mechanisms of autophagy with clinal trials to investigate autophagy drugs (Table 2).

**TABLE 2:** Autophagy enhancer in kidney disease.

Agent	Targets	Kidney disease	References
Rapamycin	mTOR-dependent	Cisplatin - induced AKI	Wang et al. (2020)
Luteolin		Inorganic mercury-induced kidney injury	Xu et al. (2021)
Triptolide		Diabetic Renal Fibrosis	Li et al. (2017)
Dapagliflozin		High-fat-diet fed rats accompanied by decreased kidney autophagy	Jaikumkao et al. (2021)
Everolimus		Renal transplantation	Pascual et al. (2018)
Cyclocarya paliurus triterpenic acids		Kidney injury in diabetic rats	Zhang et al. (2019)
Rhein		adenine (Ade)-induced renal tubular injury	Tu et al. (2017)
Trehalose	Nrf2	Cadmium-induced kidney injury	(Yaribeygi et al. (2019), Atwood et al. (2020), Fan et al. (2020)
	Atg12-5 complexes	polycystic kidney	Atwood et al. (2020)
	Rab9a		
	TFEB	Cisplatin-induced acute kidney injury	Zhu et al. (2020)
Geniposide	Ras/Raf/MEK/ERK	Lipopolysaccharide (LPS)-caused murine kidney podocyte MPC5 apoptosis and autophagy	Li et al. (2019b)
Sarsasapogenin	GSK3β	Diabetic nephropathy	Li et al. (2021b)
Sulforaphane	Nrf2	Obesity-related glomerulopathy	Lu et al. (2020)
Salvianolic acid B	Sirt1	renal fibrosis rats	He et al. (2020)
Hyperoside	AMPK-ULK1	D-galactose induced renal aging	Liu et al. (2018)
Metformin	Sirt1/FoxO1	Diabetic nephropathy	Xu et al. (2020a)

## 5 Conclusion and perspectives

Currently, the research of autophagy in kidney is at early stage. Worthy of note, a multitude of studies have yielded many significant advances both in autophagy and kidney diseases during various pathological states. Despite these encouraging findings, the functions of autophagy are abstruse and many unanswered questions remain. It is indisputable that autophagy is increasingly considered to be a therapeutic target in kidney diseases such as AKI, CKD, aging, and renal fibrosis. Mount of signal pathways participate in autophagy; conversely, dysregulated autophagy gives rise to the pathogenesis of kidney diseases. Thus, investing and clarifying the precise mechanisms of autophagy in different kidney diseases is not only a potential therapeutic target but also is scientifically intriguing and clinically relevant.

## Abbreviations

AKI, acute kidney disease; ALR, autophagic lysosome reformation; AMPK, AMP-activated protein kinase; ATGs, Autophagy-related genes; CMA, chaperone-mediated autophagy; CKD, chronic kidney disease; DN, diabetic nephropathy; EMT, epithelial-mesenchymal transition; ER, endoplasmic reticulum; ESRD, end-stage renal disease; FOXOs, forkhead transcription factors; GPCRs, G protein coupled receptors; IR, ischemia-reperfusion; IRI, ischemia-reperfusion injury; LC3, light chain 3; NAD+, nicotinamide adenine dinucleotide; mTOR, mammalian target of rapamycin; PAR2, protease activated receptors 2; PDK1, phosphoinositide-dependent-protein kinases 1; PKC, Protein kinase C; PI3Ks, Phosphatidylinositol 3-kinases; PTEN, phosphatase and tensin homolog deleted from chromosome 10; RCC, renal cell carcinoma; RTKs, receptor tyrosine kinases; SAKI, sepsis-induced acute kidney injury; T2DM, type 2 diabetes mellitus.
